# Polycaprolactone in Bone Tissue Engineering: A Comprehensive Review of Innovations in Scaffold Fabrication and Surface Modifications

**DOI:** 10.3390/jfb15090243

**Published:** 2024-08-24

**Authors:** Hsin-Yu Liang, Wei-Keung Lee, Jui-Tsen Hsu, Jie-Yu Shih, Tien-Li Ma, Thi Thuy Tien Vo, Chiang-Wen Lee, Ming-Te Cheng, I-Ta Lee

**Affiliations:** 1 School of Dentistry, College of Oral Medicine, Taipei Medical University, Taipei 11031, Taiwan; b202112060@tmu.edu.tw (H.-Y.L.); b202112063@tmu.edu.tw (J.-T.H.); b202112080@tmu.edu.tw (J.-Y.S.); 2 Department of Physical Medicine and Rehabilitation, Taoyuan General Hospital, Ministry of Health and Welfare, Taoyuan 33004, Taiwan; wk5113@gmail.com; 3 School of Dental Technology, College of Oral Medicine, Taipei Medical University, Taipei 11031, Taiwan; tlma113005@tmu.edu.tw; 4 Faculty of Dentistry, Nguyen Tat Thanh University, Ho Chi Minh 70000, Vietnam; vtttien@ntt.edu.vn; 5 Department of Nursing, Division of Basic Medical Sciences, and Chronic Diseases and Health Promotion Research Center, Chang Gung University of Science and Technology, Chiayi 61363, Taiwan; cwlee@gw.cgust.edu.tw; 6 Department of Respiratory Care, Chang Gung University of Science and Technology, Chiayi 61363, Taiwan; 7 Department of Orthopaedic Surgery, Chang Gung Memorial Hospital, Chiayi 61363, Taiwan; 8 Department of Orthopedic Surgery, Taoyuan General Hospital, Ministry of Health and Welfare, Taoyuan 33004, Taiwan; 9 College of Medicine, National Yang Ming Chiao Tung University, Taipei 11221, Taiwan; 10 Department of Biomedical Engineering, Chung Yuan Christian University, Taoyuan 32023, Taiwan; 11 Department of Orthopedic Surgery, Taoyuan General Hospital, Ministry of Health and Welfare, Sinwu Branch, Taoyuan 32748, Taiwan

**Keywords:** bone tissue engineering, polycaprolactone, 3D printing, scaffold modification, surface coatings, hydroxyapatite, nanomaterials, graphene oxide

## Abstract

Bone tissue engineering has seen significant advancements with innovative scaffold fabrication techniques such as 3D printing. This review focuses on enhancing polycaprolactone (PCL) scaffold properties through structural modifications, including surface treatments, pore architecture adjustments, and the incorporation of biomaterials like hydroxyapatite (HA). These modifications aim to improve scaffold conformation, cellular behavior, and mechanical performance, with particular emphasis on the role of mesenchymal stem cells (MSCs) in bone regeneration. The review also explores the potential of integrating nanomaterials and graphene oxide (GO) to further enhance the mechanical and biological properties of PCL scaffolds. Future directions involve optimizing scaffold structures and compositions for improved bone tissue regeneration outcomes.

## 1. Introduction

### 1.1. Overview of Bone Tissue Engineering (BTE)

BTE represents a promising field that aims to develop strategies for bone repair and regeneration. Essential properties of materials used in BTE include biocompatibility, biodegradability, osteoinductivity, osteoconductivity, controlled drug release, responsiveness to physiological conditions, and adequate mechanical strength to support bone function during healing [1,2]. Commonly utilized materials in BTE include polycaprolactone (PCL), β-tricalcium phosphate (β-TCP), calcium sulfate (CS), and bicalcium phosphate (BCP). These materials are chosen for their ability to assist bone tissue recovery, restore mechanical properties, and promote the natural processes of bone regeneration and healing [3]. An ideal scaffold for BTE must degrade at a rate that matches the formation of new bone tissue when implanted in vivo [1]. This synchronization ensures that the scaffold provides temporary support and gradually transfers the load to the regenerating bone. Porous scaffolds, in particular, have garnered attention for their innovative therapeutic potential. Their interconnected pore structures facilitate cell attachment, proliferation, and differentiation, which are crucial for effective bone regeneration. Additionally, these scaffolds allow for nutrient and waste exchange, enhancing the overall healing process [3,4,5]. Recent advancements in BTE have focused on improving scaffold design and material properties. For instance, incorporating growth factors and signaling molecules within the scaffold matrix can enhance osteoinductivity, further promoting bone tissue formation. Furthermore, the development of composite materials that combine the beneficial properties of different biopolymers and ceramics has shown promise in creating scaffolds with superior mechanical and biological performance. Overall, BTE is advancing toward more sophisticated and clinically applicable solutions for bone repair. By leveraging the inherent properties of biomaterials and incorporating bioactive components, researchers aim to develop scaffolds that not only support bone healing but also actively participate in the regenerative process, ultimately leading to better clinical outcomes for patients with bone defects or injuries. The continuous innovation in scaffold technology and material science holds the potential to transform the future of bone tissue engineering [3].

**Figure 1:**
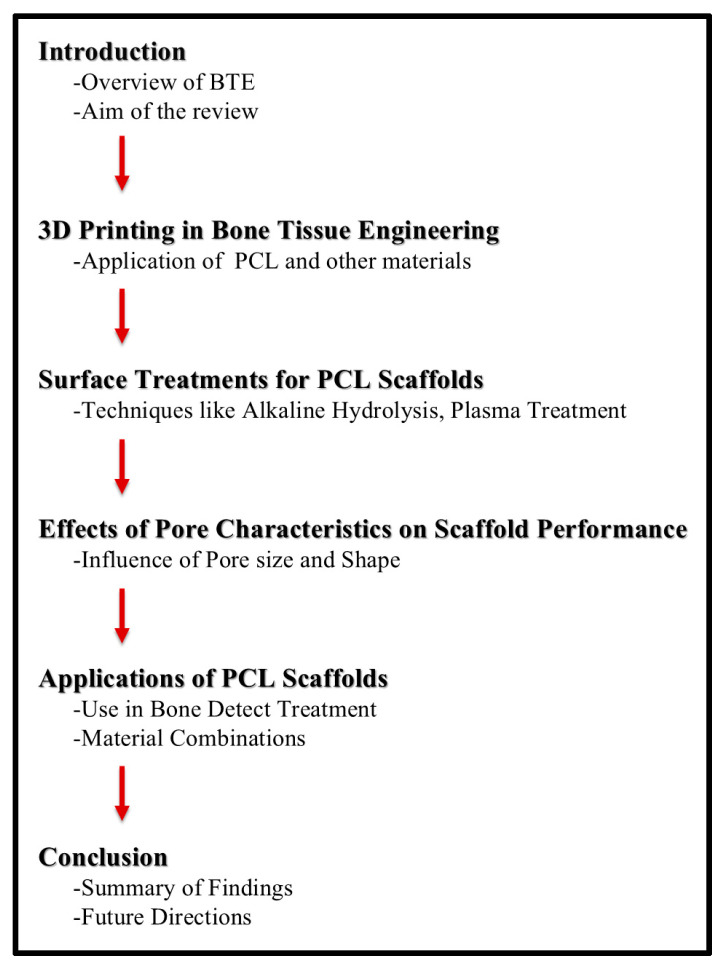
Structure of the narrative review. This flowchart outlines the key sections of the narrative review, including the introduction, 3D printing in bone tissue engineering, surface treatments for PCL scaffolds, effects of pore characteristics on scaffold performance, applications of PCL scaffolds, and the conclusion. Each section highlights the main topics discussed, providing a clear overview of the content and organization of the review.

In this review, we aim to provide a comprehensive overview of the current advancements in scaffold technology, particularly focusing on innovations in 3D printing, scaffold surface modifications, and material compositions (Figure 1). The research strategy involved a systematic search of peer-reviewed articles from databases such as PubMed, Scopus, and Web of Science using specific keywords related to ‘bone tissue engineering’, ‘scaffold modifications’, ‘3D printing’, and ‘polycaprolactone scaffolds’. Articles published in the last 10 years were prioritized to capture the most recent advancements. Inclusion criteria included studies that specifically addressed scaffold modifications for bone tissue regeneration, while studies unrelated to bone or focusing solely on other tissue engineering fields were excluded. Although this is a narrative review, these inclusion and exclusion factors ensured that only relevant, high-quality studies were reviewed to provide a detailed analysis of the topic.

### 1.2. Three-Dimensional (3D) Printing in Bone Tissue Engineering

Mesenchymal stem cells (MSCs) play a pivotal role in bone regenerative medicine due to their significant differentiation potential. MSCs can differentiate into various cell types, excluding hematopoietic cells. While MSCs contribute to tissue repair, they lack the capability to reconstruct an entire organ. Primarily sourced from bone marrow, MSCs can also be derived from adipose tissue, adult muscle, deciduous tooth pulp, and corneal stroma, among other tissues [6]. The use of MSCs in combination with 3D-printed scaffolds holds great promise for enhancing bone regeneration and repair, offering a versatile approach to addressing various orthopedic and dental challenges. By exploring the synergistic potential of 3D printing technologies and MSCs, researchers aim to develop more effective and customized solutions for bone tissue engineering. The integration of advanced materials and stem cell technology continues to pave the way for innovative therapeutic strategies, promising improved outcomes for patients suffering from bone-related ailments.

Three-dimensional printing has become a crucial method in bone tissue engineering due to its precise and repeatable fabrication capabilities. This technique is often integrated with bone tissue engineering to produce rapid and custom bone scaffolds using a variety of materials [7,8]. Numerous 3D printing techniques, such as bioprinting, fused deposition modeling (FDM), gas foaming, stereolithography, electrospinning, and powder metallurgy, are utilized to create these scaffolds [1,9]. Notably, the surfaces of these printed scaffolds retain the chemical properties of the original materials, ensuring biocompatibility and functionality [8]. Material extrusion 3D printing, in particular, shows immense promise across various tissue engineering applications, including bone, cartilage, skin, and vascular tissues [7]. In the context of the fused deposition modeling (FDM) technique, thermoplastic polymers such as PCL, polylactide (PLA) [10], poly (lactic acid) (PLLA) [11], and poly (vinyl alcohol) (PVA) [12] are frequently used. These biodegradable polymers are highly valued for their biocompatibility, allowing them to be safely implanted into the body where they can gradually degrade and be absorbed over time. The porous structures of these scaffolds mimic the extracellular matrix of human bone, facilitating cell growth and tissue formation [13]. Among these materials, PCL is particularly preferred for medical applications, especially in fabricating bone scaffolds, due to its FDA approval. PCL is a synthetic, biocompatible, non-cytotoxic, low-cost, and biodegradable polymer that is extensively used for its bioabsorbable properties, making it a prime candidate for medical use [14]. Additionally, PCL has a relatively low melting point of around 60 degrees Celsius, which is advantageous for 3D printing compared with other polymers like PLA [15]. However, despite its beneficial properties, PCL’s hydrophobic nature poses limitations in vivo, such as reduced cell attachment, affinity, and proliferation [16]. To address these challenges, PCL can be blended with other polymers or ceramics or surface-modified to enhance its properties for specific applications. Incorporating additional features while maintaining biocompatibility is essential for optimizing 3D-printed PCL scaffolds for bone tissue engineering.

## 2. Surface Treatments for PCL Scaffolds

Although PCL exhibits numerous advantageous properties, its inherent hydrophobic nature and that of its copolymers present significant challenges for their expanded use in biomedical applications [17]. This hydrophobicity can negatively impact cell attachment, proliferation, and overall biocompatibility, limiting the effectiveness of PCL-based scaffolds in tissue engineering. To address these issues, various surface modification techniques have been explored to enhance the hydrophilicity and bioactivity of PCL. One common approach to improve PCL’s surface properties involves physical treatments such as plasma treatment, which introduces polar functional groups onto the polymer surface, thereby increasing its wettability and promoting better cell attachment and proliferation [18]. Another method is the chemical modification of PCL surfaces through processes like hydrolysis or aminolysis, which also add functional groups that enhance the material’s hydrophilicity and biocompatibility. Additionally, blending PCL with more hydrophilic polymers or incorporating bioactive molecules such as peptides, proteins, or growth factors onto the scaffold surface can significantly improve its cellular interactions. For instance, grafting hydrophilic polymers like polyethylene glycol (PEG) onto PCL can enhance its surface properties and improve cell adhesion and growth [18]. Similarly, coating PCL scaffolds with extracellular matrix proteins like collagen or fibronectin has been shown to enhance cellular responses and tissue integration. Through these surface modifications, critical properties such as hydrophobicity, degradation rates, and poor cell adhesion can be significantly altered, thereby overcoming the material’s inherent limitations and expanding its applicability in various biomedical fields [18]. These advancements in material science are crucial for the development of more effective and reliable scaffolds for tissue engineering and regenerative medicine. By leveraging these innovative strategies, researchers continue to push the boundaries of what is possible with PCL, paving the way for new and improved applications in bone, cartilage, and other tissue engineering domains. This ongoing research underscores the importance of surface modification techniques in optimizing the performance of biomaterials for medical use.

### 2.1. Surface Coatings

To enhance the properties of PCL for bone regeneration applications, it is advantageous to combine it with other biomaterials that possess crystal structures and chemical properties similar to the inorganic components of bone tissue. Examples of such biomaterials include hydroxyapatite (HA), bioactive glass, and tricalcium phosphate (TCP) derivatives. These materials can effectively mimic the natural bone matrix and contribute to improved scaffold performance.

β-TCP is a well-known synthetic material renowned for its osteoconductive and osteoinductive properties. Its resorption is mediated by cellular activity, making it an excellent choice as a bone graft substitute. β-TCP facilitates the release of calcium and phosphorus ions that play a crucial role in promoting cell proliferation and differentiation [19]. A study by Ghezzi et al. demonstrated that PCL/β-TCP composite scaffolds loaded with up to 70% β-TCP exhibit structural properties suitable for bone substitution, highlighting the material’s effectiveness in bone regeneration [20]. Incorporating additional bioactive materials, such as bioglass, into β-TCP/BG scaffolds has been shown to significantly improve their mechanical properties. For instance, scaffolds with 20% bioglass have been reported to achieve a compressive strength of 8.34 MPa and an elastic modulus of approximately 200 MPa [21]. Similarly, the inclusion of 30% β-TCP in PCL scaffolds notably enhances the compressive modulus, resulting in superior mechanical performance compared with scaffolds with lower β-TCP content [22]. Research by Ngo et al. indicated that adding 20% β-TCP increased the hardness and Young’s modulus of the scaffolds, thereby improving their resistance to load deflection [23]. However, some studies have reported contrasting results where increasing β-TCP content led to a decrease in the average compressive modulus [24]. Moreover, Wang et al. observed that in PCL/β-TCP scaffolds, the stress value increased with the addition of carbon nanotubes (CNTs), with scaffolds containing 0.3% CNT exhibiting the highest stress values [25]. The efficacy of these scaffolds is also assessed through various biological indicators, including cell viability, alkaline phosphatase (ALP) activity, osteogenic gene expression, and mineral deposition. ALP, a key phenotypic marker for osteoblasts, is used to evaluate the early differentiation of MG-63 cells. Increased ALP activity indicates progressive osteoblast differentiation and maturation of the extracellular matrix with collagen. For instance, Javkhlan et al. reported significantly higher ALP activity on PCL/β-TCP scaffolds compared with pure PCL scaffolds after 14 days of culture [26]. Wang et al. observed ALP staining (blue-purple) on both thick and thin coarse fibers of the PT scaffold after 7 days of induction, indicating enhanced osteogenic activity [27].

The degradation rate of scaffolds is critical for balancing mechanical support, tissue growth, and biocompatibility. Ideally, a scaffold should degrade at a rate that matches the formation of new bone tissue. If the scaffold degrades too quickly, it may fail to support the new tissue adequately. Conversely, a scaffold that degrades too slowly may obstruct natural bone formation and remodeling processes. Studies have shown that PCL scaffolds with added β-TCP degrade faster than pure PCL scaffolds, while those with 20% β-TCP maintain sufficient mechanical strength to support human cancellous bone even after 6 weeks of degradation [28]. Future research should focus on optimizing the mechanical properties of composite scaffolds by improving their structure and material distribution. Additionally, investigating the in vivo and in vitro degradation kinetics of these materials will provide valuable insights into their long-term performance and effectiveness. By tailoring the scaffold’s composition and architecture, it is possible to develop more efficient and reliable solutions for bone regeneration applications. This will likely involve the exploration of new materials, innovative fabrication techniques, and comprehensive testing to ensure that the scaffolds can meet the demands of clinical use. Furthermore, combining PCL with other biocompatible materials and incorporating advanced surface modification techniques will continue to play a pivotal role in overcoming the current limitations and enhancing the overall performance of these scaffolds in bone tissue engineering.

### 2.2. Alkaline Hydrolysis

Plasma treatment and alkaline hydrolysis are considered effective methods for altering the chemical properties of PCL scaffolds. Both techniques not only modify the surface characteristics but also introduce new functional groups, providing an oxidizing environment. This is particularly advantageous compared with laser treatment. However, it is important to note that an excessive treatment time can compromise the mechanical properties of the scaffolds, making the selection of an appropriate duration crucial. One study highlighted the ability of alkaline hydrolysis to uniformly treat the entire scaffold, which is particularly beneficial for 3D-printed scaffolds compared with plasma treatments [29].

The commonly used hydrolysis method is alkaline treatment with sodium hydroxide (NaOH). Hydroxide ions (OH^−^) from NaOH react with the ester bonds in PCL, leading to their hydrolysis into carboxyl (COOH) and hydroxyl (OH) groups [30,31]. An increase in these newly formed functional groups can enhance the hydrophilicity and wettability of the PCL scaffold surface, thereby improving the interaction between cells and the scaffold, which ultimately promotes cell adhesion and growth. Scaffolds treated with NaOH show increased surface roughness compared with untreated scaffolds. The extent of surface modification is influenced by the shape and size of the pores in the scaffolds. In a study by Yaseri et al., it was found that scaffolds with smaller pore sizes and triangular geometry exhibited rougher surfaces and deeper pores at various NaOH concentrations. Specifically, scaffolds treated with 1 M NaOH exhibited optimal surface roughness and pore structures while maintaining intact filament shapes [1]. Additionally, significant morphological changes were noted at higher NaOH concentrations (1 M and 2 M) and with extended treatment durations [32]. Regarding mechanical properties, both studies concluded that hydrolysis resulted in a reduction in the mechanical performance of the PCL samples. However, despite this decline, the treated scaffolds maintained adequate mechanical integrity and were still suitable for facilitating bone regeneration at low load-bearing sites. Therefore, while increased surface roughness and porosity enhance cellular proliferation, they also reduce mechanical strength. A study investigating the surface modification of PCL/HA scaffolds using oxygen plasma and NaOH found that alkaline treatment exposed HA particles in the scaffolds more effectively than oxygen plasma treatment. This exposure significantly enhanced the proliferation and differentiation of human dental pulp stem cells [33]. Another study showed that the optimal biological and mechanical performance of NaOH-treated scaffolds occurs at an intensity level of approximately x ~ 65. At this level, the scaffolds demonstrate the best balance of in vitro results and mechanical properties [34]. The study found that a short, five-minute soak in sodium hydroxide increased the fiber diameter, reduced pore size, improved hydrophilicity, and enhanced cell attachment and proliferation [35].

When comparing acid and base treatments of the scaffold, it is observed that hydrochloric acid exposure leads to significant overall degradation, while NaOH treatment results in more superficial degradation. Regarding surface charge changes, NaOH treatment causes a greater increase in surface charge, which becomes more pronounced with higher alkali concentrations. Concerning mechanical degradation and molecular weight change, treating the scaffolds with acid markedly diminishes their mechanical strength and considerably decreases their molecular weight. While the physical characteristics of the scaffolds are reduced following alkali treatment, the change in molecular weight is minimal. From a physiological perspective, the degradation characteristics observed under acid catalysis are more representative of physiological degradation. Additionally, the apatite-like layer formed on the alkali-treated surface is superior in terms of the surface coating [36]. In summary, alkaline hydrolysis with NaOH is a scalable and cost-effective method to optimize PCL scaffolds, improving their interaction with cells and ensuring their suitability for tissue engineering applications. The use of this method allows for significant enhancement of the scaffold’s properties, making it a valuable approach for future research and development in the field of bone tissue engineering.

### 2.3. Plasma Treatment

Plasma treatment is an advanced surface modification technique designed to improve the hydrophilicity of electrically conductive scaffolds and to create biomaterials with physically roughened nanostructured surfaces. This process involves exposing the material to a plasma environment, which is an ionized gas consisting of free electrons, ions, and neutral particles. Plasma treatment can significantly enhance the surface properties of materials by introducing various functional groups, such as hydroxyl (OH), carboxyl (COOH), and carbonyl (C=O) groups, thereby increasing the material’s surface energy and wettability [37].

Compared with using natural-based hydrophilic polymers such as gelatin, collagen, and elastin to address hydrophobicity issues, plasma treatment offers several significant advantages. First, plasma treatment induces minimal damage to the PCL scaffold, preserving its original mechanical properties and structural integrity. This is crucial for maintaining the scaffold’s strength and functionality in various biomedical applications, particularly at load-bearing sites [38]. Second, plasma treatment is an environmentally friendly process. Unlike methods that rely on chemical modifications using potentially harmful solvents, plasma treatment operates without the need for toxic chemicals, making it a safer and more sustainable option. This ensures that cellular activities are not adversely affected, thereby maintaining the biocompatibility of the treated scaffolds [38]. Furthermore, plasma treatment is highly efficient and can be precisely controlled to achieve the desired surface modifications. By adjusting parameters such as plasma type, treatment duration, and power, researchers can tailor the surface characteristics to enhance hydrophilicity, improve cell adhesion, and promote tissue integration. This level of control is often difficult to achieve with natural-based polymers, which can vary in composition and properties. Another advantage of plasma treatment is its versatility. It can be applied to a wide range of materials, including polymers, metals, and ceramics, making it a universal technique for surface modification. This adaptability allows for the development of customized scaffolds for specific tissue engineering applications, from bone regeneration to cardiovascular implants [38]. In addition, plasma treatment has been shown to create surfaces with nanoscale roughness, which can mimic the natural extracellular matrix and enhance cellular responses. This nano-topography can improve protein adsorption and cell signaling, leading to better cell proliferation and differentiation. Such enhancements are particularly beneficial for scaffolds used in regenerative medicine where the interaction between the scaffold and surrounding tissues is critical for successful integration and function [38]. Overall, plasma treatment stands out as a superior method for enhancing the hydrophilicity of PCL scaffolds. Its ability to preserve mechanical properties, environmental friendliness, precise control, versatility, and effectiveness in creating bioactive surfaces make it an invaluable tool in the field of tissue engineering and regenerative medicine.

Plasma treatment significantly alters the chemical and physical properties of PCL scaffolds, bringing about a range of improvements. First, the surface of the scaffold becomes notably coarser after plasma treatment, which enhances its texture and promotes better cell adhesion. Despite this increase in surface roughness, the scaffold retains its original pore size due to its high stability in thermal and chemical environments, ensuring that its structural integrity is maintained [37]. Second, the wettability of the PCL scaffold undergoes a dramatic transformation. Prior to plasma treatment, the water contact angle of the PCL scaffold is higher than 100 degrees, indicating a hydrophobic surface. After plasma treatment, the water contact angle approaches 0 degrees, signifying that the scaffold has become highly hydrophilic. This enhanced hydrophilicity is crucial for improving the interaction between the scaffold and biological tissues, facilitating better cell attachment and proliferation [38]. Third, the oxygen-to-carbon (O/C) ratio of PCL surfaces increases upon exposure to oxygen plasmadue to the surface oxidation of the treated scaffolds. This increase in the oxygen component significantly enhances the hydrophilic properties of the scaffolds, thereby improving initial cell adhesion. Habibovic and colleagues have shown that this surface oxidation plays a critical role in enhancing the scaffold’s biocompatibility and promoting cellular activities [38]. Additionally, it is important to ensure that the mechanical properties of the PCL scaffold are not adversely affected by plasma treatment. Measurements of the compressive strength have shown that there are no statistically significant differences in the Young’s moduli of the PCL scaffolds before and after plasma treatment, with values ranging from 24.9 to 25.2 MPa. This indicates that plasma treatment does not compromise the mechanical integrity of the scaffold, which is essential for its use in load-bearing applications [38].

In terms of biocompatibility, the plasma-treated PCL scaffold demonstrates significantly higher cell viability compared with untreated scaffolds. In experiments conducted by Sajjad Shafei, the plasma-treated PCL scaffold exhibited approximately 350 cells/mm^2^, whereas the untreated PCL scaffolds showed merely 120 cells/mm^2^. This substantial increase in cell density indicates that plasma treatment greatly enhances the scaffold’s ability to support cell growth and proliferation, making it more suitable for bone regeneration applications [37]. Based on the data mentioned above, it is evident that plasma treatment offers significant advantages for improving the properties of PCL scaffolds. The enhanced surface roughness, increased hydrophilicity, improved oxygen content, maintained mechanical properties, and superior biocompatibility—all contribute to making plasma-treated PCL scaffolds highly effective for use in bone regeneration and other tissue engineering applications. In dental applications, the use of lasers, such as the Erbium:Yttrio-Aluminum-Granate (Er:YAG) laser, is prevalent due to their precision and ability to preserve surrounding tissues. However, laser treatments can alter the surface characteristics and mechanical properties of scaffold materials. For instance, laser irradiation may increase surface roughness, thereby improving cell attachment and proliferation. However, it may also introduce localized heating effects, which could affect the scaffold’s structural integrity. Recent studies have shown that Er:YAG laser can effectively reduce the microbial population on treated surfaces without compromising the scaffold’s biocompatibility, making them a valuable tool in scaffold-based therapies [39]. This highlights the importance of considering laser effects in the design and application of scaffolds for dental tissue engineering.

### 2.4. Aminolysis

In various experiments, researchers employ aminolysis using different amino acids, such as ethylenediamine and gelatin, to enhance the biological activity of PCL scaffolds and increase cell attachment sites [40]. Regardless of the specific amino acids used, the primary goal is to improve the scaffold’s bioactivity, thereby promoting better cellular interactions and tissue integration. To prepare the PCL scaffold for aminolysis, it is first immersed in isopropanol for twelve hours. This step ensures that any residual impurities are removed. After thorough washing with deionized water, the scaffold is dried at 30 degrees Celsius overnight. This preparation process is crucial for ensuring the scaffold’s surface is clean and ready for the subsequent aminolysis treatment. During the aminolysis procedure, the scaffold membrane is immersed in a solution containing the chosen amino acids for a specified duration. This process facilitates the introduction of active amino groups onto the PCL nanofibers through aminolysis reactions. The active amino groups significantly enhance the scaffold’s surface properties by providing more sites for cell attachment, which is essential for improving cell adhesion and proliferation. Following aminolysis treatment, the scaffold is thoroughly washed with deionized water multiple times to remove any unreacted amino acids and other residues. This step is critical to ensuring that the scaffold is free from any contaminants that could affect its performance. The treated scaffold is then dried in a vacuum oven at 30 degrees Celsius overnight, which helps to stabilize the newly introduced functional groups on the scaffold surface [41]. The introduction of amino groups through aminolysis not only improves the hydrophilicity of the PCL scaffold but also enhances its ability to support cell growth. The amino groups provide additional binding sites for cells, leading to improved cell attachment and proliferation. This modification is particularly beneficial for applications in tissue engineering where the interaction between the scaffold and the surrounding biological environment is crucial for successful tissue regeneration. Furthermore, aminolysis can be tailored to introduce various bioactive molecules onto the scaffold surface, further enhancing its functionality. By carefully selecting the type and concentration of amino acids used in the aminolysis solution, researchers can fine-tune the scaffold’s properties to meet specific requirements for different biomedical applications. This versatility makes aminolysis a valuable tool in the development of advanced biomaterials for regenerative medicine. In summary, aminolysis treatment is a highly effective method for improving the biological activity of PCL scaffolds. By introducing active amino groups onto the scaffold surface, this technique enhances cell attachment and proliferation, making the scaffolds more suitable for tissue engineering applications. The detailed preparation and treatment process ensures that the scaffolds are optimized for their intended use, ultimately contributing to the success of tissue regeneration efforts.

Aminolysis treatment brings several changes to PCL scaffolds, resulting in a range of improvements that make them more suitable for biomedical applications compared with untreated scaffolds. The surface of aminolysis-treated scaffolds becomes noticeably rougher, and the nanofibers tend to fuse together. Despite these surface changes, the overall morphological features of aminolysis-treated PCL scaffolds do not exhibit remarkable differences compared with untreated scaffolds. This indicates that while aminolysis alters the surface texture to enhance cell interaction, it does not drastically change the scaffold’s overall structure, maintaining its integrity for practical applications [32]. Aminolysis also affects the mechanical properties of PCL scaffolds. The Young’s modulus of treated PCL scaffolds decreases slightly from 12.3 ± 1.6 MPa to about 11.8 ± 2.7 MPa. This modest reduction suggests that while aminolysis does impact the mechanical properties, it does not cause severe damage. The scaffolds retain sufficient mechanical integrity, making them suitable for various biomedical applications, particularly in low-load-bearing environments [41,42]. The elemental composition of the PCL scaffold surface is significantly altered by aminolysis. Energy dispersive X-ray spectroscopy (EDX) analysis shows that as the immersion time in amino acids increases, the percentage of carbon decreases while the percentages of oxygen and nitrogen increase. This shift indicates the formation of amide bonds on the surface of PCL scaffolds, resulting from the decay of ester bonds and the emergence of amine groups. These chemical modifications enhance the scaffold’s surface properties, making it more conducive to cell attachment and proliferation [32]. The wettability of the PCL scaffold surface improves significantly after aminolysis. Static contact angle measurements reveal a reduction in the water contact angle, indicating that the surface becomes less hydrophobic. This improvement in hydrophilicity is expected, given the presence of free amino groups, which are hydrophilic. Moreover, the water contact angle decreases by approximately 25 degrees for every 15 min of aminolysis, demonstrating the effectiveness of this treatment in enhancing surface wettability [40]. Aminolysis treatment significantly enhances the biocompatibility of PCL scaffolds. Studies show a substantial increase in cell proliferation on aminolysis-treated scaffolds compared with untreated ones. Interestingly, further increasing the concentration of the amino solution or extending the treatment duration does not significantly affect cell proliferation, suggesting an optimal level of modification is sufficient for promoting cell growth. In vitro experiments reveal that aminolysis modifications create an optimal surface for cell attachment, spreading, and proliferation, making these scaffolds highly suitable for tissue engineering applications [5,43]. Based on the aforementioned research, aminolysis emerges as a favorable option for improving the properties of PCL scaffolds for tissue engineering applications. The enhancements in surface roughness, mechanical integrity, elemental composition, wettability, and biocompatibility contribute to the scaffold’s effectiveness in supporting tissue regeneration.

## 3. Effects of Pore Characteristics on Scaffold Performance

### 3.1. Effect of Pore Size

In bone tissue engineering, the pore size of a scaffold significantly influences key factors such as cell viability, cell attachment, and the initial attachment of cells. There is no universal standard for the optimal pore size, as the most suitable pore size can vary depending on factors like the ratio between cell and pore size or the humidity of the implantation environment. Both large and small pore sizes have their respective advantages and disadvantages [1].

First and foremost, cell attachment is critical for the success of bone tissue engineering. Studies indicate that cells favor rough scaffold surfaces because they provide more prominent recognition sites for anchoring. Before treatment with NaOH solution, the scaffold surfaces are often too smooth for effective cell adhesion. However, after NaOH treatment, the PCL scaffold becomes rougher due to the penetration of the solution, which enhances cell attachment. The impact of pore size on this process is significant. Smaller pores are more susceptible to high concentrations of NaOH solution because of the slow diffusion rate and increased exposure resulting from a larger surface area [1]. Larger pore sizes, on the other hand, facilitate higher fluid velocities and rapid diffusivity, which can result in insufficient time for cells to attach to the scaffold surfaces. Scaffolds with a pore size of 700 μm tend to exhibit smoother surfaces and fewer cells compared with those with 500 μm pores. The mechanical properties of scaffolds are also affected by the pore size. An increase in the pore size can lead to a slight decrease in compressive strength as larger pores might reduce the compressive modulus. It has been found that the mechanical strength of scaffolds with smaller pores is similar to that of cancellous bone (5–10 MPa) [44].

Additionally, reducing the scaffold pore size (from 700 μm to 500 μm) decreases the spacing between filaments while increasing the number of filaments, which demonstrates that smaller pore sizes are better for cell growth [1]. Studies have also shown that protein adsorption, which contributes to cell attachment, is enhanced in scaffolds with small pore sizes (50–500 nm PLLA scaffolds) [4]. Despite the drawbacks of large pore sizes in terms of cell attachment and mechanical properties, they offer some advantages. Large pore sizes ensure adequate cellular nutrition, while small pore sizes may face the risk of pore occlusion. Preventing pore occlusion over time requires not only larger pore sizes but also higher diffusivity in the center of the scaffold [1]. In summary, the characteristics of different pore sizes in scaffolds significantly affect their performance in bone tissue engineering. While smaller pore sizes generally enhance cell attachment and mechanical strength, larger pore sizes facilitate better nutrient diffusion and prevent pore occlusion. Balancing these factors is crucial for optimizing scaffold design for specific tissue engineering applications.

### 3.2. Effect of Pore Shapes

In bone tissue engineering, the shape and arrangement of pores within scaffolds play crucial roles in determining their effectiveness. Common pore shapes include cube, triangle, and honeycomb [1,45]. While data on honeycomb-shaped pores is limited, the characteristics of triangle- and cube-shaped pores are well documented. Scaffolds with small triangle-shaped pores possess several notable features. First, NaOH treatment significantly impacts filament thickness and pore size [1]. As the NaOH concentration increases, the porous architecture and dimensions change, leading to a decline in structural integrity and cell viability [1]. Additionally, the surface roughness increases, resulting in higher permeation and lower diffusion rates. The triangular pore structure is similar to cancellous bone, providing excellent mechanical strength [1]. It also improves compressive properties slightly and offers numerous crossing points for cell fibers to generate [46]. However, cells still develop better in their native morphology. In contrast, scaffolds with cube-shaped pores exhibit different characteristics. The NaOH concentration does not significantly influence the pore size and filament thickness as it does with triangular pores [1]. Cube-shaped pores result in smoother scaffold surfaces. Cell viability and ingrowth are enhanced with small cubic pores [1]. A greater number of concave surfaces in the cube geometry supports better cell bridging and growth, but this also leads to faster pore occlusion [1]. The arrangement of pore shapes is typically categorized into three zones: irregular-, regular-, and no-pore zones. These variations arise due to differences in viscosities, extrusion velocities, flow rates, and strut diameters. Irregular-pore zones occur when both extrusion velocity and strut diameter are low due to the humping phenomenon of slow extrusion velocity [7]. No-pore zones emerge when both values are high, causing the strut diameter to exceed the intended thickness [7]. Regular-pore zones appear in the medium range between the no-pore and irregular-pore zones. Interestingly, the addition of silver does not alter the pore morphology or microstructures [3]. Understanding these arrangements allows for adjustments in the 3D printing process to improve scaffold quality further.

In addition to pore shapes and arrangements, the channel structure within scaffolds is also a critical factor. Scaffolds can be designed as non-channeled, straight-channeled, or branched channeled. There is no significant difference in the compressive strength and degradation rate among these three types of scaffolds. Non-channeled scaffolds allow for cell infiltration, but straight channels enhance cell penetration into deeper pores more effectively [47]. Branched channels offer the most advantages. Fluid passes through branched channels much quicker, which is essential for facilitating nutrient and waste transport [47]. The distribution of branched channels significantly influences cell locations, with most cells infiltrating the channel zones. High porosity can be achieved in branched channeled scaffolds without notably sacrificing compressive strength [47]. This structure also promotes rapid vascularization, which is crucial for bone tissue regeneration [47]. In summary, both pore shapes and channel structures are vital for optimizing scaffolds for bone tissue engineering. Triangle-shaped pores provide excellent mechanical strength and surface roughness, while cube-shaped pores enhance cell viability and growth. Regularly arranged pore zones improve scaffold quality, and branched channels significantly boost fluid transport and vascularization, making them highly promising for bone tissue regeneration.

## 4. Applications of PCL Scaffolds

PCL scaffolds have become a cornerstone in tissue engineering, particularly for their role in the restoration of injured bone tissue. Their wide usage can be attributed to their favorable properties such as biodegradability, biocompatibility, and mechanical strength, which make them suitable for supporting tissue regeneration. Furthermore, PCL scaffolds are often combined with other materials, including bioactive ceramics, growth factors, and stem cells, to enhance their curative effects. These combinations can significantly improve osteoconductivity, promote cell adhesion and proliferation, and accelerate the healing process. The incorporation of additional materials into PCL scaffolds aims to mimic the natural bone environment more closely, providing a synergistic effect that enhances overall tissue repair and regeneration. Consequently, PCL scaffolds represent a versatile and effective approach in the field of bone tissue engineering, offering promising outcomes for patients with bone injuries.

### 4.1. Diseases Commonly Treated with PCL Scaffolds

The PCL scaffold is designed not only to restore injured bone tissue and its mechanical properties but also to promote natural bone regeneration and healing progression [3]. Its biocompatibility, biodegradability, non-toxicity, plasticity, and suitable mechanical properties make the PCL scaffold an excellent substitute for functional tissue [48]. Consequently, the primary application of PCL scaffolds is in bone defect management and substitution [47]. PCL scaffolds are widely used in the treatment of various bone defects, including fractures, infections, bone tumors, bone loss, high-energy injuries, non-unions of bone, mal-unions of bone, and osteonecrosis or avascular necrosis [6,47,49]. These scaffolds benefit a broad range of orthopedic surgeries by providing structural support and promoting bone regeneration. Additionally, PCL scaffolds are utilized in some long-term implantable devices, offering innovative solutions for sustained structural integrity and biological function [3]. Beyond bone defect management, PCL scaffolds have diverse applications, including wound dressings, fixation implants, drug delivery systems, and various dentistry applications [3,6]. The versatility of PCL scaffolds extends to specialized designs that can control the release rate of antibacterial agents, thereby preventing infections and enhancing healing processes [3]. This feature is particularly beneficial in creating scaffolds tailored for specific clinical needs, such as sites prone to bacterial contamination. Although some applications remain challenging, the clinical use of PCL scaffolds has already demonstrated significant benefits. The ability to replace bone in a short period accelerates healing processes, thus improving the quality of life for patients. For instance, in cases of complex fractures or large bone defects, the rapid integration and osteoconductive properties of PCL scaffolds facilitate quicker recovery times and better clinical outcomes [6]. In addition to orthopedic applications, the role of PCL scaffolds in dentistry has shown promising results. They are used in periodontal regeneration, maxillofacial reconstruction, and as carriers for drug delivery in dental treatments. These scaffolds support the growth of new tissue and help restore the function and aesthetics of dental structures. Overall, PCL scaffolds are a versatile and effective solution in regenerative medicine. Their use in treating a variety of conditions underscores their importance in modern medical practices. As research continues, the potential applications of PCL scaffolds are likely to expand, bringing further improvements to patient care and surgical outcomes.

### 4.2. Materials Combined with PCL Scaffolds

Various materials are used in combination with scaffolds to enhance their effectiveness in tissue engineering. Each material offers unique benefits and poses certain limitations, which are summarized in Table 1. Below is a detailed description of these materials.

#### 4.2.1. Hydrogel

Hydrogels are highly valued in bone tissue engineering due to their appropriate pore dimensions, which facilitate osteointegration, osteoconduction, and degradation, allowing for effective bone ingrowth [6]. One of the significant advantages of hydrogels is their versatility in size, shape, and form, enabling the customization of scaffolds to meet specific clinical needs [6]. By altering the physical and chemical properties of hydrogels, the stability of the implant can be adjusted, providing tailored solutions for various medical applications [6]. Hydrogels are particularly promising for promoting osteoporotic bone repair due to their ability to support new bone formation. In addition to the advantages and challenges highlighted, recent studies have shown that hydrogels, when integrated with PCL scaffolds, can significantly improve osteoconductivity and provide a more favorable microenvironment for bone regeneration [50,51]. These studies emphasize the importance of optimizing the physical and chemical properties of hydrogels to achieve better stability and functionality in clinical applications. However, a notable drawback is that the degradation process of hydrogels can produce harmful by-products, which may pose risks to the surrounding tissues [2]. Additionally, when hydrogels swell, they can negatively impact compatibility and mechanical stability. This swelling can increase pressure on the surrounding tissues, potentially causing damage and discomfort [2]. These limitations restrict the application of hydrogels in osteoporotic bone regeneration where mechanical stability and biocompatibility are crucial. Despite these challenges, ongoing research aims to optimize hydrogels to minimize harmful by-products and control swelling behavior. Innovations such as incorporating bioactive molecules or nanoparticles into hydrogels are being explored to enhance their properties and broaden their clinical applications. The potential for hydrogels to be fine-tuned for specific medical scenarios makes them a promising area of study in regenerative medicine. Moreover, hydrogels can be combined with other materials to create composite scaffolds that leverage the strengths of each component. For instance, integrating hydrogels with PCL can enhance the mechanical stability and biocompatibility of the scaffold, providing a more robust solution for bone tissue engineering. These composite materials can offer the benefits of hydrogels’ osteoconductive properties while mitigating some of their inherent disadvantages. In summary, while hydrogels present certain challenges in bone tissue engineering, their versatility and potential for customization make them valuable materials. By addressing the issues related to harmful by-products and swelling, hydrogels could play a significant role in advancing bone regeneration techniques, particularly in the treatment of osteoporotic conditions.

#### 4.2.2. Nanomaterials

Nanomaterials offer several advantages in the field of bone tissue engineering, including versatility, effectiveness, rapid action, adjustability, and minimal invasiveness [2]. Their small size allows them to be integrated into various scaffold designs, enhancing the overall functionality of the scaffold. The active chemical properties of nanomaterials make it easy to modify and integrate biological factors, such as growth factors and proteins, which can promote cell differentiation and tissue regeneration [2]. Inorganic nanoparticles, in particular, can significantly improve the mechanical properties of scaffolds. By incorporating these nanoparticles into the scaffold matrix, the strength, stiffness, and durability of the scaffold can be enhanced, making it more suitable for load-bearing applications [2]. This ability to reinforce scaffolds is especially valuable in orthopedic applications where mechanical integrity is crucial. Recent research has demonstrated the effective integration of nanomaterials with PCL scaffolds, leading to enhanced mechanical properties and improved cellular responses [52,53]. The addition of nanomaterials like hydroxyapatite nanoparticles has been particularly beneficial in reinforcing the structural integrity of scaffolds, making them more suitable for load-bearing applications. However, the use of nanomaterials is not without challenges. One of the primary concerns is their potential cytotoxicity. The small size and high reactivity of nanoparticles can lead to adverse biological reactions, which may affect cellular health, particularly in long-term applications [2]. The cytotoxic effects can vary depending on the type of nanomaterial, its concentration, and the duration of exposure. Therefore, careful consideration and thorough testing are required to ensure the safe use of nanomaterials in clinical settings. Despite these challenges, ongoing research is focused on mitigating the cytotoxic effects of nanomaterials. Strategies such as surface modification, encapsulation, and controlled release mechanisms are being explored to reduce toxicity while maintaining the beneficial properties of nanomaterials. Additionally, the development of biocompatible and biodegradable nanomaterials is a promising avenue that could enhance their safety profile for long-term use. Nanomaterials can also be combined with other materials to create composite scaffolds that leverage their unique properties. For instance, combining nanomaterials with hydrogels or PCL can create scaffolds that are not only mechanically robust but also biologically active. This approach can provide a synergistic effect, improving both the structural and functional aspects of the scaffold. In summary, nanomaterials hold great potential in bone tissue engineering due to their versatility, ability to enhance mechanical properties, and capacity for biological modification. However, their cytotoxicity remains a significant concern that needs to be addressed through innovative research and development. With continued advancements, nanomaterials could play a crucial role in the future of regenerative medicine, offering new solutions for complex medical challenges.

#### 4.2.3. Graphene Oxide (GO)

GO is renowned for its exceptional properties, making it a valuable material in bone tissue engineering. GO boasts a high specific surface area, excellent thermal conductivity, and impressive mechanical resistance. These characteristics enable GO to reinforce the structural integrity of scaffolds, enhancing their performance in load-bearing applications. In biological fluids, GO demonstrates greater solubility, higher reactivity, and stability, which are critical for maintaining its functionality within the body [54]. Its ability to generate reactive oxygen species further enhances its utility, providing antitumor and antimicrobial properties that can prevent infections and promote healthier tissue environments [54]. This dual functionality makes GO an attractive option for applications requiring both structural support and biological activity. Moreover, the incorporation of GO into scaffolds significantly improves their modulus of elasticity, making the scaffolds more flexible and better able to mimic the mechanical properties of natural bone [54]. This improvement is crucial for the development of scaffolds that can withstand the dynamic stresses and strains experienced in the body, particularly in orthopedic applications. GO’s high reactivity allows for easy functionalization with various biological molecules, such as proteins, peptides, and growth factors. This ability to be chemically modified enhances GO’s compatibility with biological tissues and promotes cell adhesion, proliferation, and differentiation. Researchers are exploring ways to harness these properties to create advanced scaffolds that can support and enhance the natural healing processes. Incorporating GO into PCL scaffolds has shown promising results in enhancing not only the mechanical properties but also the biological interactions, leading to improved cell adhesion and proliferation [55,56]. These studies highlight the potential of GO to significantly elevate the performance of PCL-based scaffolds in both orthopedic and neurological applications. However, while GO offers numerous benefits, there are challenges that must be addressed. The biocompatibility and potential cytotoxicity of GO are areas of active research. Ensuring that GO-based scaffolds are safe for long-term implantation involves thorough in vitro and in vivo testing. Additionally, the scalability of producing GO with consistent quality and properties is crucial for its widespread clinical application. In summary, GO is a highly promising material in the field of bone tissue engineering due to its excellent mechanical properties, high reactivity, and biological activity. Its ability to enhance the modulus of elasticity and provide antimicrobial and antitumor effects makes it an invaluable component in developing advanced scaffolds. Continued research and development will be essential to fully realizing the potential of GO in clinical applications, ensuring safety and efficacy for long-term use.

#### 4.2.4. HA

HA is a crucial material in bone tissue engineering due to its ability to significantly enhance the properties of scaffolds used for bone regeneration. HA improves the pore architecture, ensuring that the scaffold has an optimal structure for cell infiltration and tissue growth. Its biodegradability allows the scaffold to gradually dissolve, making way for new bone formation without the need for surgical removal [1,57]. Additionally, HA contributes to the mechanical properties of the scaffold, providing the necessary strength and stability to support bone tissue in load-bearing applications. The bioactivity of HA enhances bone-bonding abilities, promoting the integration of the scaffold with the host bone tissue and ensuring a strong and stable connection [1,57]. Incorporating HA nanoparticles into a PCL matrix results in a composite material that offers superior performance in bone tissue engineering. This combination leverages the osteoconductive properties of HA, which guide the growth of new bone cells along the scaffold, facilitating more effective and efficient bone regeneration [57]. The PCL matrix provides a flexible and supportive framework, while the HA nanoparticles enhance the overall bioactivity and mechanical strength of the scaffold. This synergistic effect makes HA-PCL composites an excellent choice for bone substitution, particularly in complex and critical-sized defects where robust support and rapid healing are required. The combination of HA with PCL scaffolds not only enhances mechanical properties but also significantly improves osteoinductivity, making these composites highly effective for bone tissue engineering [58,59]. This synergistic effect is particularly beneficial in complex bone regeneration scenarios where robust support and rapid healing are required. Moreover, HA’s chemical similarity to natural bone mineral makes it highly biocompatible, reducing the risk of adverse reactions and promoting faster healing. The presence of HA in the scaffold also stimulates the differentiation of stem cells into osteoblasts, the cells responsible for bone formation, further enhancing the regenerative process. Researchers are continually exploring ways to optimize the integration and performance of HA within various scaffold matrices to maximize its benefits in clinical applications. In summary, HA significantly enhances the effectiveness of scaffolds in bone tissue regeneration by improving pore architecture, biodegradability, mechanical properties, and bioactivity. Its incorporation into PCL matrices creates a superior composite material with excellent osteoconductive properties, making it a valuable component in the development of advanced bone graft substitutes. Continued research and innovation in this area hold promise for further improving the outcomes of bone regeneration therapies.

## 5. Conclusions

This review has provided a comprehensive overview of recent advancements in scaffold fabrication and surface modifications for bone tissue engineering. Significant progress has been made in enhancing the properties of PCL-based scaffolds through techniques such as 3D printing, surface treatments, and the incorporation of nanomaterials and graphene oxide. These innovations have the potential to significantly improve scaffold conformation, cellular behavior, and mechanical performance, making them promising candidates for future clinical applications. However, it is important to acknowledge several limitations of this review. The scope was confined to studies published within the last ten years, which may have excluded earlier foundational work that could still be relevant to current research. Additionally, the focus on PCL-based scaffolds, due to their widespread use in bone tissue engineering, may limit the applicability of the findings to other biomaterials. Moreover, while the review discusses various surface modification techniques, it does not extensively cover in vivo studies, which are crucial for understanding the long-term biocompatibility and clinical effectiveness of these scaffolds. The potential ethical and regulatory challenges associated with the clinical translation of these advanced scaffolds are also beyond the scope of this review. Despite these limitations, the advancements discussed offer promising directions for the future of bone tissue engineering. Future research should aim to address the identified limitations by expanding the range of materials studied, incorporating more in vivo investigations, and exploring the ethical and regulatory implications of scaffold-based therapies. By overcoming these challenges, the development of more effective and clinically applicable scaffolds will be possible, ultimately leading to improved outcomes for patients with bone defects or injuries.

## Figures and Tables

**Table 1:** Summary of advantages and disadvantages of various materials used in bone tissue engineering.

Materials	Advantage	Disadvantage
Hydrogel [2,6,50,51]	- Appropriate pore dimension - High plasticity - Increased osteoporotic bone repair efficiency - Improved osteoconductivity	- Reduced compatibility - Reduced mechanical stability - Hurting surrounding tissues
Nanomaterials [2,52,53]	- Versatility - Effectiveness - Speed - Adjustability - Minimal invasiveness - Active chemical properties - Enhanced mechanical properties - Improved cellular responses	- Cytotoxicity
Graphene oxide [54,55,56]	- Enhancement of the modulus of elasticity - Good thermal conductivity - Mechanical resistance - High specific surface area - Enhanced mechanical properties - Improved cell adhesion and proliferation	
Hydroxyapatite [1,57,58,59]	- Increased pore architecture - Improved biodegradability - Enhanced bone-bonding abilities - Increased bioactivity - Improved osteoinductivity - Enhanced mechanical properties	

## Data Availability

Not applicable.
